# Propionyl-L-Carnitine Enhances Wound Healing and Counteracts Microvascular Endothelial Cell Dysfunction

**DOI:** 10.1371/journal.pone.0140697

**Published:** 2015-10-16

**Authors:** Maria Giovanna Scioli, Pietro Lo Giudice, Alessandra Bielli, Valeria Tarallo, Alfonso De Rosa, Sandro De Falco, Augusto Orlandi

**Affiliations:** 1 Department of Biomedicine and Prevention, Anatomic Pathology, University of Tor Vergata, Rome, Italy; 2 Sigma-Tau S.p.A., Pomezia, Rome, Italy; 3 Consiglio Nazionale delle Ricerche, Institute of Genetics and Biophysics "A. Buzzati Traverso", Naples, Italy; 4 Policlinic of Tor Vergata, Anatomic Pathology, Rome, Italy; IDIBAPS - Hospital Clinic de Barcelona, SPAIN

## Abstract

**Background:**

Impaired wound healing represents a high cost for health care systems. Endothelial dysfunction characterizes dermal microangiopathy and contributes to delayed wound healing and chronic ulcers. Endothelial dysfunction impairs cutaneous microvascular blood flow by inducing an imbalance between vasorelaxation and vasoconstriction as a consequence of reduced nitric oxide (NO) production and the increase of oxidative stress and inflammation. Propionyl-L-carnitine (PLC) is a natural derivative of carnitine that has been reported to ameliorate post-ischemic blood flow recovery.

**Methods and Results:**

We investigated the effects of PLC in rat skin flap and cutaneous wound healing. A daily oral PLC treatment improved skin flap viability and associated with reactive oxygen species (ROS) reduction, inducible nitric oxide synthase (iNOS) and NO up-regulation, accelerated wound healing and increased capillary density, likely favoring dermal angiogenesis by up-regulation for iNOS, vascular endothelial growth factor (VEGF), placental growth factor (PlGF) and reduction of NADPH-oxidase 4 (Nox4) expression. In serum-deprived human dermal microvascular endothelial cell cultures, PLC ameliorated endothelial dysfunction by increasing iNOS, PlGF, VEGF receptors 1 and 2 expression and NO level. In addition, PLC counteracted serum deprivation-induced impairment of mitochondrial β-oxidation, Nox4 and cellular adhesion molecule (CAM) expression, ROS generation and leukocyte adhesion. Moreover, dermal microvascular endothelial cell dysfunction was prevented by Nox4 inhibition. Interestingly, inhibition of β-oxidation counteracted the beneficial effects of PLC on oxidative stress and endothelial dysfunction.

**Conclusion:**

PLC treatment improved rat skin flap viability, accelerated wound healing and dermal angiogenesis. The beneficial effects of PLC likely derived from improvement of mitochondrial β-oxidation and reduction of Nox4-mediated oxidative stress and endothelial dysfunction. Antioxidant therapy and pharmacological targeting of endothelial dysfunction may represent a promising tool for the treatment of delayed wound healing or chronic ulcers.

## Introduction

Physiological wound healing is a highly organized dynamic process requiring tight temporal and spatial coordination of various events and signaling networks aimed to provide a protective barrier against further external stimuli or infections [1,2]. Wound healing involves the interaction between extracellular matrix, connective tissue contraction, multiple soluble mediators, blood cells, keratinocytes, endothelial cells, and angiogenesis for tissue remodeling and repair [1]. In normal conditions, endothelium provides a semi-permeable membrane for the transfer of nutrients and retrieval of waste products, regulates monocyte adhesion and subsequent infiltration at the site of injury, promotes vasodilatation and inhibits inflammation, and thrombosis [3]. During wound healing, angiogenesis leads to the formation of a dense network of small blood vessels in the granulation tissue. Endothelial dysfunction contributes to microangiopathy that impairs cutaneous microvascular blood flow, hypoxia and accelerated inflammation, causing delayed healing or chronic wounds [4,5]. The management of wounds presents a significant burden to healthcare services, consuming a large amount of resources [6]. Experimental data suggest that wound healing associates with endothelial aberrations suggestive of localized dysfunction [3]. Animal models of impaired cutaneous wound healing revealed a reduction in nitric oxide (NO) production, which associates with reduced collagen accumulation and wound breaking strengths [7]. Reduction in cutaneous blood flow, oxygen tension, abnormal angiogenesis and increased levels of matrix metalloproteinases (MMPs) and reactive oxygen species (ROS) further support the hypothesis that endothelial cell dysfunction is responsible for impaired wound repair [3]. Vascular inflammation and increased production of ROS play a pivotal role in endothelial dysfunction [8,9]. ROS are produced by several enzyme systems, including NADPH oxidase (Nox), xanthine oxidase, endothelial nitric oxide synthase, lipoxygenases and myeloperoxidase [10]. Although all those enzymes can potentially contribute to the oxidative stress, Nox is the predominant source of ROS in the vasculature and Nox4 the major endothelial isoform [11]. The cellular fueling system, in particular the mitochondrial β-oxidation pathway, is a target for various noxious stimuli, including oxidative stress [12]. Inflammatory stimuli can induce the increase of cellular oxidative stress driven by increasing mitochondrial oxidative stress and Nox activity [13,14], with a consequent mitochondrial dysfunction and impairment of β-oxidation [15–16].

Endothelial dysfunction is also accompanied withan increased expression of cell adhesion molecules, such as intercellular adhesion molecule-1 (ICAM-1), vascular cell adhesion molecule-1 (VCAM-1), and inflammatory cytokine secretion [17,18]. Adhesion molecules are critical because they mediate inflammatory cell recruitment into the subendothelial space [19]. Clinical reports described that therapy with antioxidants and free radical scavengers are efficacy in patients with endothelial dysfunction [20,21].

Propionyl-L-carnitine (PLC) is an ester of L-carnitine, required for the transport of fatty acids into the mitochondria [22]. L-carnitine is an endogenous substance that acts as a carrier for fatty acids across the inner mitochondrial membrane necessary for subsequent β-oxidation and ATP production [23]. PLC was also documented to be an antioxidant agent, so protecting tissues from oxidative damage [8,24,25]. In particular, PLC has been documented to be capable to reduce membrane lipid peroxidation and the effects of hypoxia in cardiomyocytes [26], endothelial dysfunction in ischemic rabbit limbs [24] and in human inflammatory bowel diseases [25]. A preliminary study reported PLC as clinically effective in the healing of arterial or venous cutaneous chronic ulcers in 14 of 18 vasculopathic patients refractory to all other forms of therapy. The beneficial effect was associated with an ameliorated blood flow recovery [27]. In order to document if beneficial effect of PLC on healing process is exclusively related to blood flow recovery or also depends from other mechanisms, we tested the efficacy of PLC in two models of cutaneous wound healing in rat: the skin flap [28] and full-thickness skin wound [29]. Since PLC was also documented to ameliorate serum-deprived dysfunction of human umbilical vein endothelial cells (HUVECs) [8,24], we aimed to investigate if beneficial effects of PLC can be extended to cutaneous microcirculation. Our in vivo studies and in vitro data by using human dermal microvascular endothelial cells (HMVECs) strongly support that, in addition to its vasodilatative capacity, PLC accelerates dermal wound healing through its beneficial anti-oxidant effects on microvascular endothelial dysfunction and reparative angiogenesis.

## Materials and Methods

### Ethics statement

Animal protocols were submitted to the Italian Ministry of Health; Department of Public Health, Animal Health, Nutrition and Food Safety. The Ministry of Health approved this study, as per article 7 of the 116/92 Ministerial Decree. All procedures were performed under anesthesia, and all efforts were made for minimize potential pain, suffering, or distress, and enhance animal welfare, according to the Three Rs (3Rs) (EU Directive 2010/63/EU).

Human leukocytes were isolated from peripheral blood of volunteersunder written informed consent and in accordance with the guidelines of the Declaration of Helsinkiand the local Ethics Committee on Human Research of Tor Vergata University that approved this study.

### Animals

We used 13–14 weeks old male Wistar rats (200–225 g; Harlan Nossan S.r.l., Correzzana, MI Italy) allowed to acclimate in controlled environmental conditions (temperature 22 ± 2°C, relative humidity 55 ± 10%, 12-h light/dark cycle) for seven days prior to the experiment. Food (GLP 4RF 21, Charles River, Calco, LC Italy) and water were supplied ad libitum and health daily monitored by veterinarians.

### Skin flap

**Fig 1:**
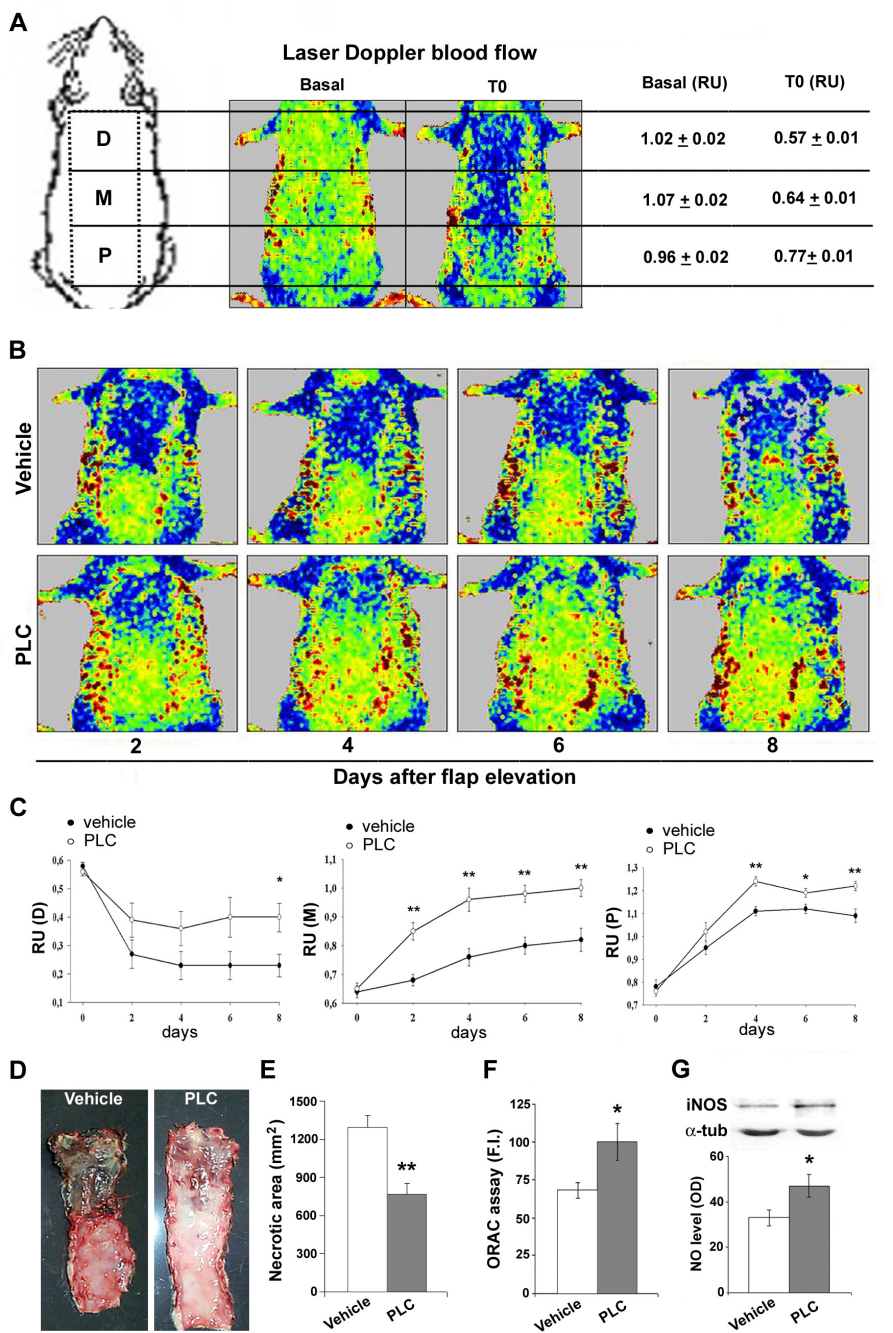
PLC ameliorates blood flow recovery. (A) Representative diagram of rat skin flap. On the left, the proximal (P), medial (M), and distal (D) segments. Center, representative laser doppler images of blood flow before (basal) and just after flap elevation (T0). Low or no blood perfusion is displayed in dark blue, and the highest perfusion level is displayed in red. Right, it was reported the means ± SEM of blood perfusion relative units (RU) in proximal, medial and distal portions of the flap before (baseline) and just after flap creation (T0). (B, C) Representative laser Doppler images of blood flow (B) and time course (C) of in skin flap in vehicle- (closed symbols) and PLC-treated (open symbols) rats. PLC was administered daily, dissolved in tap water at the dose of 100mg/kg. Points are mean ± SEM of 15 rats, in relative units of blood perfusion at 2, 4, 6 and 8 days after flap elevation. (C) Left, blood perfusion in distal portion of flap (repeated measures ANOVA: * indicates p< 0.05, PLC-treated vs vehicle-treated rats). Center, blood perfusion in medial portion of flap (ANOVA:** indicates p< 0.001,PLC-treated vs vehicle-treated rats). Right, blood perfusion in proximal portion of flap (ANOVA:* and ** indicate p< 0.05 and p< 0.001, respectively, PLC-treated vs vehicle-treated rats). (D,E) Representative images of flap skin and necrotic area measurement after 8 days. Necrotic area (black) is clearly demarcated from survival regions. (F) Values represent mean ± SEM of 15 rats. Repeated measures ANOVA: treatment, p< 0.0001; Dunnett’s test: * and ** indicate p< 0.001 and p< 0.0001, respectively. (F) Bar graph showing ROS levels, inversely proportional to fluorescence intensity (F.I), in rat skin flap. Values represent mean ± SEM of 15 rats. t-Student: * indicates p< 0.05. (G) iNOS protein expression and NO level (expressed in optical density, OD), in rat skin flap. Values represent mean ± SEM of 15 rats. t-Student: * indicates p< 0.05. Abbreviation: α-tub, α-tubulin.

Rats were anesthetized by intraperitoneal injection of pentobarbital sodium (60 mg/kg; Siegfried Ltd, Zofingen, Switzerland). The design and surgical technique for construction of the rat dorsal skin flap were previously described [28,30,31]. Briefly, dorsal surface was shaved and a 10 x 3 cm caudally based dorsal skin flap was elevated from the scapulas to the iliac crest (Fig 1A), then sutured back into its original site with stainless steel wound clips. During measurements, body temperature was maintained constant at 37°C by using a thermoregulator (BM 70002, Biomedica Mangoni, Pisa, Italy) and blood pressure checked by using a piezoelectric tail-cuff pulse transducer, as reported [32]. Rats were identified by numbers; the blood pressure technician was not aware of the experimental grouping. Special care was taken to monitor their complete recovery from anesthesia, and vital parameters. No analgesia was administered during the post-surgery recovery period. Rats were randomized into two groups (n = 15); the first group received PLC (Sigma-Tau SpA, Pomezia, Italy) at the dose of 100 mg/kg/day in the drinking water, the second group only water (vehicle). The PLC concentration in the water was adjusted on the bases of body weight and water intake, daily measured and checked for one week before the beginning of the treatment and during the experiment. Revascularization was quantified by measuring the extent of survival flap by serial assessment of blood flow [33] using a scanning laser Doppler perfusion imaging (PIM II System Laser Doppler Perfusion Imager [LDPI]; Lisca/Perimed, Järfälla-Stockholm, Sweden) before (baseline), immediately after surgery (day 0),and after 2, 4, 6, and 8 days under light anesthesia by s.c pentobarbital injection (40 mg/kg). Scanned color-coded image representing blood flow distribution displayed on the monitor were divided into three equal portions: proximal (near the iliac crest), medial at the centre of the flap, and distal at the scapula (Fig 1A). Analysis of mean perfusion was performed using the manufacturer's operational software (LDPIwin) and normalized on baseline values. After the last blood flow recording, flaps were removed by cutting the margin and animals sacrificed by a lethal dose of pentobarbital. Flap and necrotic areas were traced onto the same transparent plastic sheet and planimetrically measured in blind (SigmaScan Pro5 Images Measurement Software), as reported[34]. The necrotic area in the distal portion of the skin flap was easily demarcated by gross observation [30].

### Full-thickness skin wound

After anesthesia by intraperitoneal injection of pentobarbital sodium (60 mg/kg) and shaving, a full-thickness excisional wound was made by a 8 mm-punch biopsy (Acuderm, Ft. Lauderdale, FL) on the rat dorsal surface between the scapulas. Animals were then caged individually and randomized into four groups receiving, just after wound creation, PLC at doses of 30 (PLC-30, n = 15), 60 (PLC-60, n = 15) and 120 mg/kg/day (PLC-120, n = 21), or vehicle (water, n = 21). No analgesia was administered during the post-surgery recovery period. Wound closure was monitored in a blinded manner in animals gently immobilized by an operator and a second one who traced the wound outline onto acetate sheets. Tracings were digitized and areas calculated by using a computerized algorithm (SigmaScan Pro5 Images Measurement Software). Wounds were considered healed when appear macroscopically re-epithelialized, and wound surfaces smooth, homogenous in color and without residual defects [35]. At the end, animals were sacrificed by a lethal dose of pentobarbital.

### Measurement of plasma levels of Propionyl-L-carnitine

Rat blood venous samples were collected before vehicle or PLC treatment, to assess the baseline concentration, after 5 days of treatment and on day 6, after 1, 4 and 7 hours from administration. Plasma PLC concentrations were determined by high performance liquid chromatography-mass spectrometry. As shown in Table 1, during administration PLC-treated rats showed steadily higher plasma PLC levels than vehicle.

**Table 1:** Plasma Propionyl-L-carnitine (nmol/mL) levels in vehicle and PLC-treated rats.

Propionyl-L-carnitine					
	Basal	5-day	after 1h	after 4h	after 7h
**Vehicle**	0.16±0.01	0.17±0.02	0.15±0.015	0.17±0.21	0.14±0.013
**PLC**	0.15±0.00	0.54±0.06*	1.76±0.99**	0.59±0.05*	0.61±0.07*

Blood samples were obtained before (basal) and after 5 days, and 1, 4, 7h after the 6^th^ day of PLC treatment. t-Student

* and ** indicate p< 0.01 and p< 0.001. Values are expressed as mean ± SEM of 15 rats.

### Morphological and immunohistochemical investigations

Immunohistochemical study [36] was performed in paraffin-embedded sections from formalin fixed wound tissue of 6 vehicle and 6 PLC-120 rats randomly chosen and sacrificed 7 days after wound creation. After antigen retrieval, serial sections were incubated for 1 h with mouse monoclonal anti-eNOS (BD Biosciences, Franklin Lakes, NJ, USA; 1:100), anti-iNOS (Santa Cruz Biotechnology Inc., CA, USA; 1:100), anti-VEGF (Abcam, CB, UK; 1:25), anti-CD31 (Abcam; 1:100), rabbit polyclonal anti-Nox4 (Santa Cruz;1:300) and goat polyclonal anti-PlGF (Santa Cruz; 1:25), followed by secondary goat anti-mouse (Nordic Immunology, the Netherlands), goat anti-rabbit (Nordic Immunology) and donkey anti-goat antibodies (Santa Cruz), respectively. Peroxidase-Streptavidin-biotin system (Dako Corporation, CA, USA) was used for immunocomplex revelation and diaminobenzidine as final chromogen. Preliminary studies evidenced the specific immunoreaction in rat tissues (not shown). All immunostainings were performed at room temperature including positive and negative controls. For quantitative assessment of immunoreactivity, 10 randomly selected fields were analyzed within the upper region on granulation tissue [37] at 200X magnification, and the percentage of positive microvessels and staining intensity calculated using a grading system and expressed in arbitrary units, as reported [24]. Assessments were performed by two researchers in a blinded manner, with an interobserver reproducibility > 95%.

### In vitro experiences

Human dermal microvascular endothelial cells (HMVECs) and human umbilical vein endothelial cells (HUVECs; Cambrex, Milan, Italy) were grown in endothelial basal medium (EBM-2) containing 5% FBS (fetal bovine serum) and endothelial growth factor supplements (EGM-2 bullet kit, Cambrex). For PLC stimulation, cells were starved in EBM-2 containing 0.1% FBS overnight, than incubated with same medium containing PLC (1mM, stock solution dissolved in PBS) or PBS (vehicle). At different times, medium and cells were collected. Medium was frozen at -80°C and used for ELISA and NO assays. For some inhibition studies, the specific Nox4 inhibitor plumbagin [38] (10μM; Sigma-Aldrich) and L-aminocarnitine, an inhibitor of carnitine-palmitoyltransferase-2 (1μM, Sigma-Tau) were used.

### In vitro wound healing assay

HMVECs were cultured in 24-well plates at 3 x 10^5^ cells/well as confluence. The monolayers were wounded (time 0, T0) in a line across the well with a 200μl standard pipette tip. Wounded monolayers were washed twice and incubated with or without 1mM PLC in the presence of serum. The scratches were photographed in at least ten fields at T0 and after 16 hours from wound (T16) using DXM1200F digital camera (Nikon, Japan) and ACT-1 software (Nikon), and the mean percentage of scratch closure calculated. The experiment was repeated three times.

### Reverse transcriptase and Real-Time Polymerase Chain Reaction

RNA was extracted and Real-Time Polymerase Chain Reactions performed as reported [39]. Sense and anti-sense primer sequences are listed in Table 2. Results were normalized against the levels of hypoxanthine-guanine phosphoribosyl transferase (HPRT) Results were normalized on glyceraldehyde-phosphate dehydrogenase (GAPDH) as housekeeping. The results were reported as normalized fold expression of three independent experiments performed in triplicate.

**Table 2:** Real-Time Polymerase Chain Reaction primers.

Gene	Primer Sequence	Temperature of Annealing
**eNOS**	Forward 5’- GGTGATGGCGAAGCGAGTGAAGGC -3’ Reverse 5’- CTGGACTCCTTCCTCTTCCGCCGC -3’	**60°C**
**iNOS**	Forward 5’-ACCTTGGTGTTTGGGTGCCGCC-3’ Reverse 5’-GCCACCCTGTCCTTCTTCGCCTCG-3’	**60°C**
**VEGF**	Forward 5’- TCTTCAAGCCATCCTGTGTG -3’ Reverse 5’- GCCTCGGCTTGTCACATC -3’	**56°C**
**VCAM-1**	Forward 5’-GCCCATCTATGTCCCTTGCTGTG-3’ Reverse 5’- GTCAACCCAGTGCTCCCTTTGCT-3’	**58°C**
**ICAM-1**	Forward 5’- GGCTGGAGCTGT TTGAGAACACC-3’ Reverse 5’-CGGTCACACTGACTGAGGCCTTG-3’	**58°C**
**GAPDH**	Forward 5’-ACGGATTTGGTCGTATTGG-3’ Reverse 5’-GATTTTGGAGGGATCTCGC-3’	**60°C**
**PlGF**	Forward 5’-GAGGCTGTTCCCTTGCTTC-3’ Reverse 5’-GGTTACCTCCGGGGAACAG-3’	**56°C**
**Flt-1**	Forward 5’- CTTGGATTTTACTGCGGACAG -3’ Reverse 5’- GGGGACACCATTAGCATGAC -3’	**56°C**
**KDR**	Forward 5’- AGAGTGAGGAAGGAGGACGAAG -3’ Reverse 5’- GGCCAAGAGGCTTACCTAGC -3’	**56°C**

## Protein extraction and western blot analysis

The total protein extracts from cells and rat skin flaps were isolated using lysis buffer containing phosphatase and proteases. After protein content determination, proteins were blotted onto nitrocellulose membranes [40] and incubated with anti-NADPH-oxidase 4 (Nox4, Abcam), anti-iNOS (Pierce) and anti-α tubulin (Sigma Aldrich) antibodies. Specific complexes were revealed as reported [41].

### ELISA assay

PlGF protein concentration (pg/1x10^6^ cells) was assessed in culture medium at 12–24 hours by ELISA [25]. Briefly, anti-human PlGF polyclonal antibody (1 μg/ml) were used to coat 96-well plates. After an overnight incubation at 4°C, wells were washed five times with PBS containing 0.004% Tween-20 (PBT) and non-specific sites were masked with 1% BSA (3 hrs at RT). Cell culture medium (100μl) was loaded in triplicate and incubated overnight at 4°C. After washing, biotin labeled anti-human PlGF polyclonal antibody (500 ng/ml in PBET) was added to each well. Signal was revealed with a commercial kit (Vectastain elite ABC kit, Vector Laboratories, Burlingame, CA, USA). The reaction was blocked by adding 4N H_2_SO_4_ and the absorbance measured at 490 nm on a microplate reader (Biorad BenchMark, Biorad, Milano, Italy).

### Detection of intracellular reactive oxygen species

ROS levels were measured by 5-(and-6)-chloromethyl-2’,7’-dichlorodihydrofluorescein diacetate, acetyl ester (CM-H_2_DCFDA) fluorescence method (Molecular Probes, Inc., Eugene, OR, USA) as reported [24]. Briefly, after treatment cells (3x10^5^/mL) were harvested by trypsinization and incubated with 20μM CM-H_2_DCFDA for 30 min at 37°C. Dichlorofluorescein fluorescence was monitored by analyzing at least 10,000 cells in a flow cytometer (Beckman Coulter, CA, USA). We performed preliminary experiments of ROS measurement also directly on adherent cells in the 96-well plate, using a fluorescence microtiter plate reader (Beckman Coulter, CA, USA). No differences between methods were found (S1 Fig). ROS levels in rat skin flap lysates were evaluated by measuring the loss of fluorescence (after 15 min) due to peroxyl-radical presence with a fluorescence microtiter plate reader (Beckman Coulter, CA, USA) using ORAC Antioxidant Assay Kit (Zen-Bio Inc., NC, USA). The reported values are the mean of triplicate samples.

### Small interfering RNA for Nox4

A 19-nucleotide small interfering RNA (siRNA) 3’-overhanged for human Nox4 (access NM_016931) was designed by using Block-IT^TM^ RNAi Designer (Invitrogen). The siNox4 sequence was: 5’-CCUCAGCAUCUGUUCUUAA-3’, whereas a non-targeting siRNA sequence was used as control: 5’-CCTTACGTGTCTCTACTAA-3’. For transfection [8], HMVECs at 60–70% confluence were incubated with the siNox4-Oligofectamine complexes (Invitrogen) in antibiotic and serum-free medium, according to manufacture’s guidelines. Depletion of Nox4 by siRNA was confirmed by Western blot.

### Nitric oxide assay

NO level in supernatants of cells and rat skin flap lysates were measured by a Nitric Oxide Colorimetric Assay Kit (BioVision, CA, USA), according to manufacturing guidelines. Absorbance was expressed in optical density units (OD) and determined at 450 nm by using a microplate reader (Sunrise TECAN, Labx, Canada). The reported values were the mean of triplicate samples.

### Measurement of β-oxidation activity

Mitochondrial β-oxidation activity in cell lysates was evaluated by measurement of flavin adenine dinucleotide (FAD) concentration using a colorimetric assay kit (Sigma-Aldrich). After treatments, FAD content was analyzed according to manufacturer’s guidelines and the absorbance expressed in optical density units (OD) determined at 570 nm by using a microplate reader (Sunrise TECAN, Labx). All experiences were repeated in three independent experiments performed in triplicate.

### Leukocyte adhesion assay

Human leukocytes were isolated from peripheral blood of volunteers (under written consent) by using Ficoll-Paque Plus (Amersham Pharmacia Biotech, NJ, USA) according to manufacture’s guidelines and incubated with 2'7'-bis(carboxyethyl)-5(6)-carboxyfluorescein acetoxymethyl ester (2 μM; Life Technologies Europe BV, Milan, Italy) for 45 min at 37°C. Then leukocytes were counted using a hemocytometer, washed and layered (1x10^6^/mL) on endothelial cells (after treatments) for 1 h on a rocker plate. Non-adherent cells were removed during three gentle washing steps. Adhering cells were fixed in 2% glutaraldehyde and counted under a fluorescent microscope (E600 Eclipse; Nikon). Experiments were repeated in triplicate.

### Statistical Analysis

Statistical analysis were carried out using SigmaStat (Jandel Scientific, San Rafael, CA) statistical packages. Data were shown as means ± SE. In vivo experiment data were compared using ANOVA test or one-way ANOVA, followed by Dunnett’s test. In case of repeated measurements, ANOVA and Dunnett’s test were performed considering the mean effect of each dose by taking into account all time points. Data from in vitro studies were expressed as the mean of at least three different experiments plus standard error (SEM) and analyzed by Student’s t-test. Differences were considered statistically significant for p< 0.05.

## Results

### PLC treatment ameliorates skin flap blood flow recovery

No infection or mortality was observed throughout the experimental processes and rise in body weight was similar in all groups. Laser Doppler imagining of flap showed that blood flow dropped as soon as the flap was made (Fig 1A). PLC did not induce any blood pressure variation (data not shown), in accordance with the literature [32]. Serial measurements indicated that ischemia was followed by a blood flow restoration in the proximal and medial part of the flap (Fig 1B and 1C). On the contrary, in the distal part of flap skin perfusion further decreased and remained very low even on days 6 and 8 leading to necrosis (Fig 1B and 1C). PLC-treated animals showed a faster blood flow recovery of the flap (Fig 1B and 1C), and an improved viability. After 8 days, in PLC-treated rats, the necrotic skin area was less than that of vehicle-treated rats (Fig 1D and 1E, p< 0.01). In addition, PLC treatment reduced ROS production and increased NO level and iNOS expression in the skin flap (Fig 1F and 1G, p< 0.05).

### PLC ameliorates wound healing and vascular tropism in vivo and in vitro

**Fig 2:**
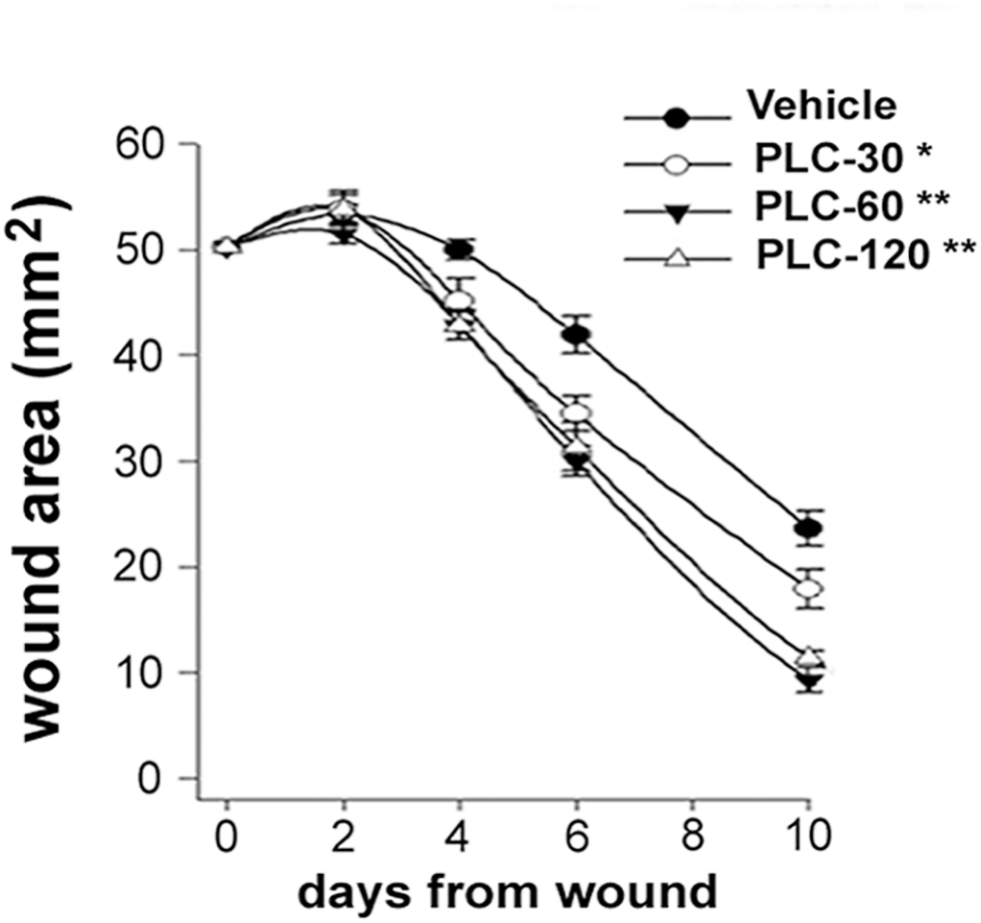
PLC accelerates full-thickness wound healing. (A) Time-course of full-thickness skin wound re-epithelialization. Statistical difference reported refers to 10 day-values. Points represent mean ± SEM of 15 rats. Repeated measures ANOVA: treatment, p< 0.0001; Dunnett’s test: * and ** indicate p< 0.001 and p< 0.0001, respectively.

**Fig 3:**
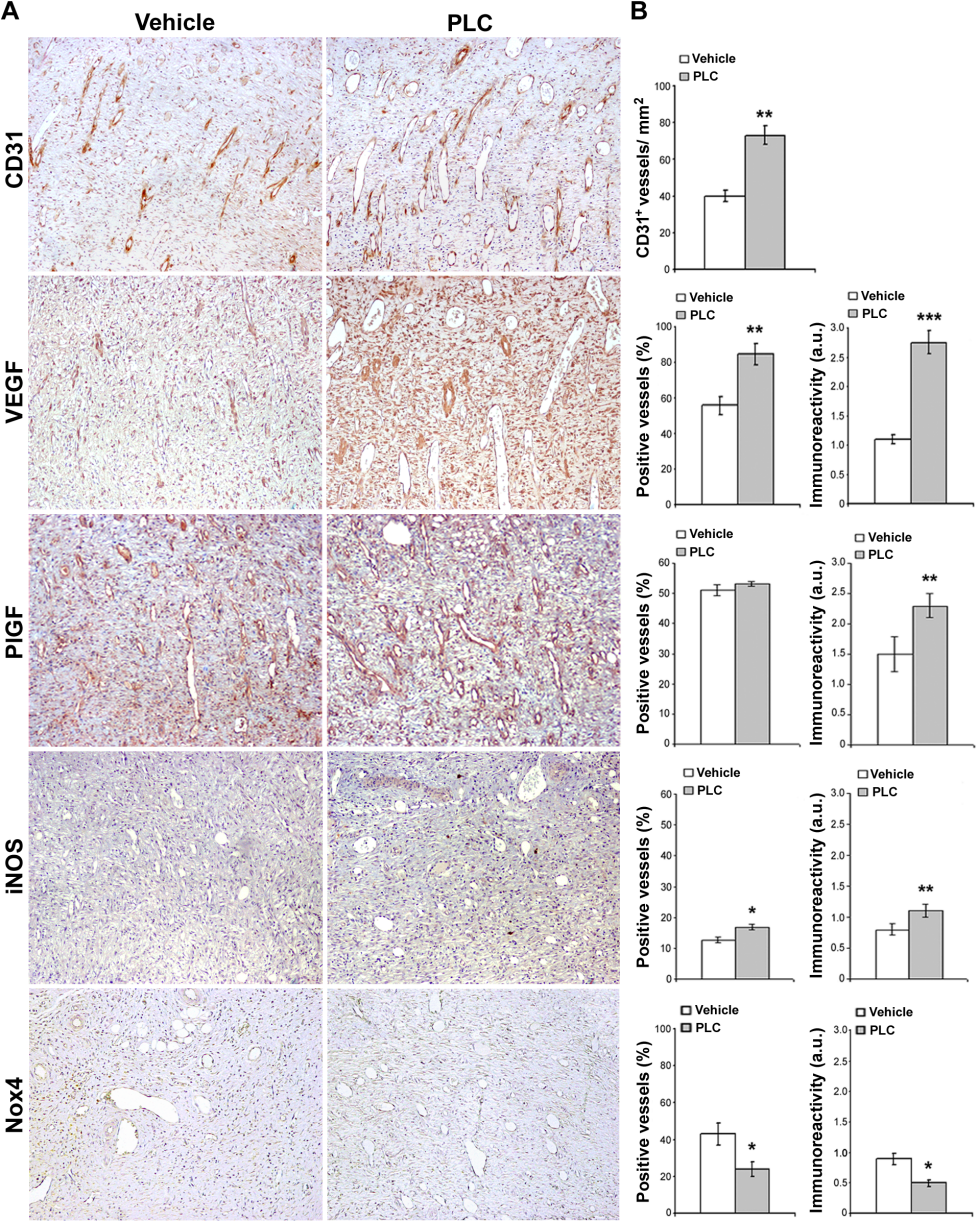
Histomorphometric analysis of rat skin wounds granulation tissue. (A) Representative CD31, VEGF, PlGF, iNOS, Nox4 immunostaining of skin granulation tissue sections in vehicle and PLC-treated rats on day 7 wounds. Magnification 100X. (B) Columns represent the percentage of immunoreactive microvessels and stain intensity calculated with a grading system in arbitrary units. Abbreviation: a.u., arbitrary units; t-Student: *, ** and *** indicate p< 0.05, p< 0.01 and p< 0.001, respectively. Values are expressed as mean ± SEM of 15 rats.

**Fig 4:**
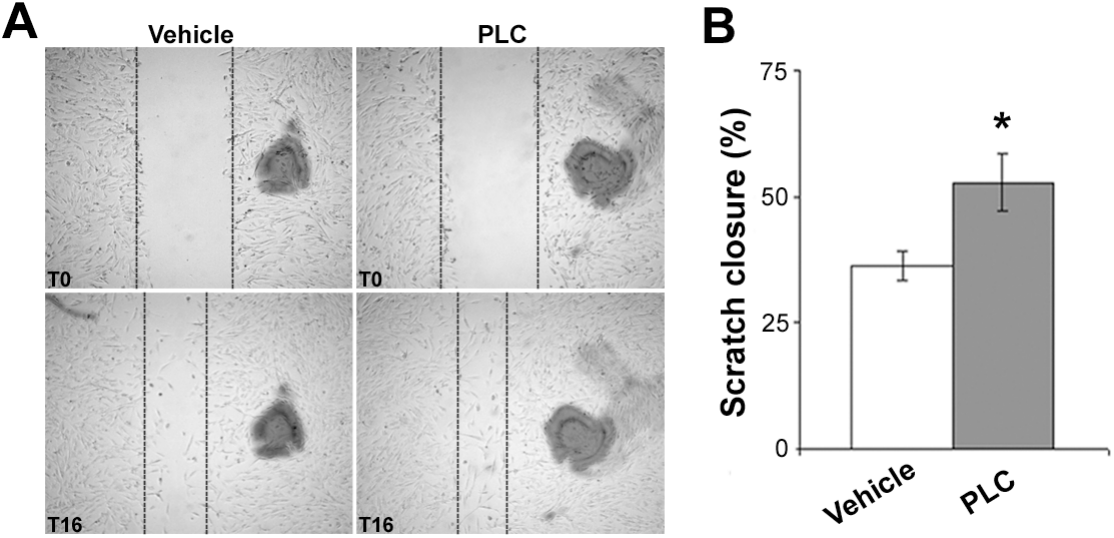
PLC accelerates experimental wound healing in vitro. (A) In vitro wound healing assay in HMVEC monolayers with or without PLC treatment (1mM) at time 0 (T0) and after 16 hours (T16). (B) Bar graph showing an increased percentage of scratch closure in PLC-treated HMVEC monolayers. Magnification 4X. t-Student: * indicates p< 0.05. Values are expressed as mean ± SEM of three separate experiments.

In PLC-treated rats, skin wounds re-epithelialized faster compared with vehicle (Fig 2) and this effect was dose-dependent. Representative microphotographs and microscopic evaluation of granulation tissue of rat skin wounds 7 days after wound are reported in Fig 3. Wounds in the upper region of both vehicle and PLC-120 treated rats showed a granulation tissue with dense CD31^+^ microvasculature (p< 0.01; Fig 3). Histomorphometric analysis documented that the percentage and signal intensity of VEGF^+^ microvessels were both increased in PLC-treated rats compared with vehicle tissue (p< 0.01 and p< 0.001, respectively); although the percentage of PlGF^+^ microvessels did not change, an increase of signal intensity was detected in PLC-treated rats (p< 0.05). Finally, PLC-treated rats showed an increase in the percentage and immunoreactivity of iNOS^+^ microvessels compared with vehicle (p< 0.05 and p< 0.01, respectively), whereas Nox4^+^ microvessels and signal intensity were reduced in PLC-treated rats (p< 0.05). Finally, as reported in Fig 4, in vitro wound healing assay showed that the percentage of scratch closure was higher in PLC-treated than in vehicle HMVEC monolayers (p< 0.05).

### PLC favor endothelial homeostasis and counteracts serum deprivation-induced dermal microvascular endothelial cell activation

**Fig 5:**
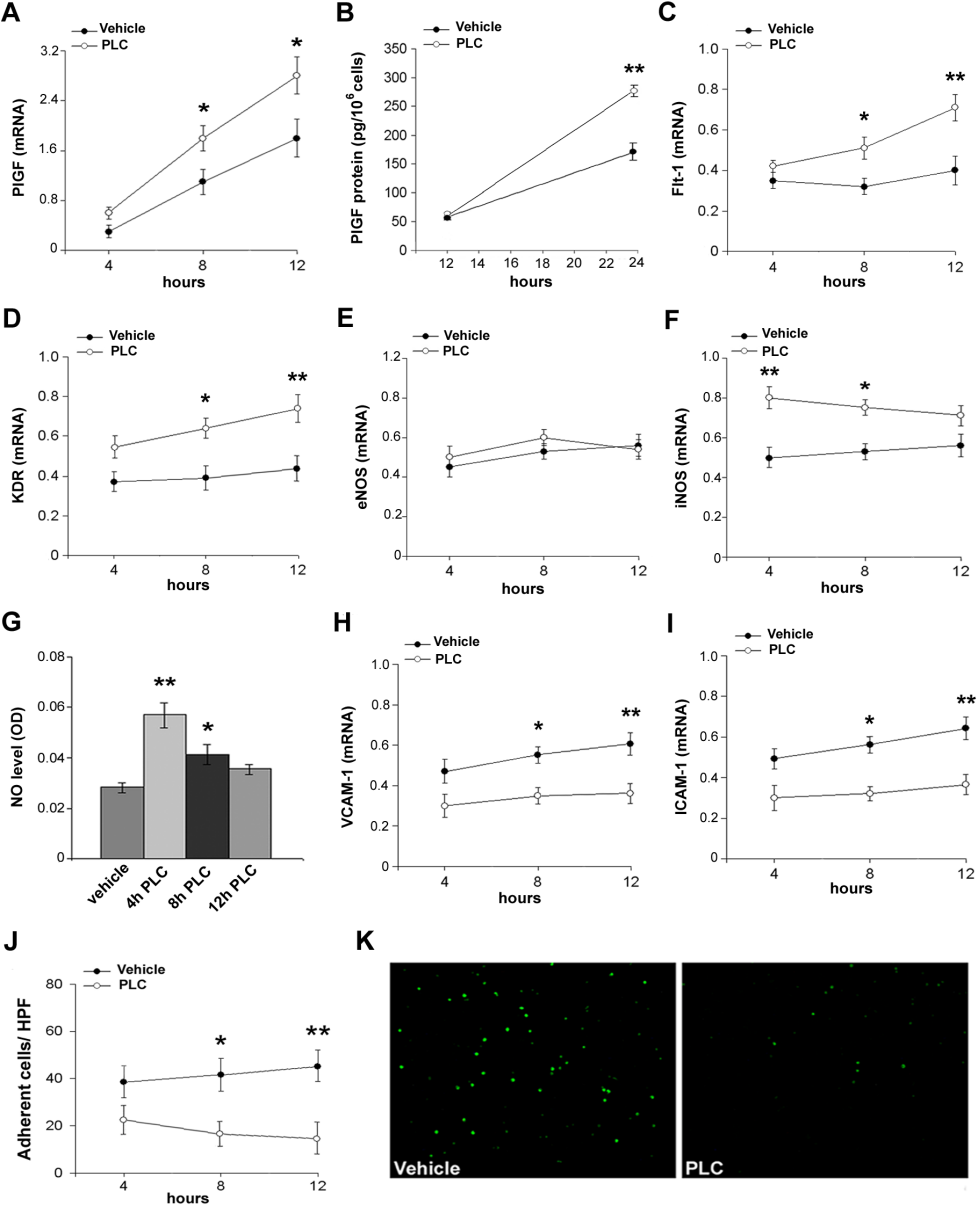
PLC ameliorates cell function in serum-deprived HMVECs. (A) Real-time PCR for PlGF mRNA in serum-deprived PBS-treated (vehicle) or PLC-treated HMVECs at different times. (B) PlGF protein concentration assessed by means of ELISA in treated cells. (C, D) Real-time PCR for Flt-1 and KDR transcripts. (E, F) Real-time PCR for eNOS and iNOS mRNA. (G) NO measurement in vehicle or PLC-treated cells at different times. (H, I) Real-time PCR for VCAM-1 and ICAM-1transcripts. (J) Leukocyte adhesion assay on vehicle and PLC-treated HMVECs and (K) representative microphotographs at 12h, magnification 100X. t-Student: * and ** indicate p< 0.05 and p< 0.01, respectively. Values are expressed as mean ± SEM of three separate experiments. Abbreviations: OD, optical density; HPF, high power field.

In order to better investigate the biomolecular targets modulated by PLC treatment in dermal microvasculature, we performed additional experiments in vitro using dermal human microvascular endothelial cells (HMVECs). As shown in Fig 5, PLC treatment caused an increase in PlGF transcript and protein expression compared to vehicle (Fig 5A and 5B), as well as a significant increase in VEGF receptor 1 (Flt-1) and VEGF receptor 2 (KDR) mRNA in HMVEC cultures (Fig 5C and 5D). Moreover, eNOS mRNA level was unchanged after PLC stimulation (Fig 5E), whereas PLC induced an increase of iNOS and NO transcript level (Fig 5F and 5G). These data support the beneficial effect of PLC on microvascular endothelial cell homeostasis. VCAM-1 and ICAM-1 expression, induced by serum deprivation-mediated endothelial activation, was counteracted by PLC treatment (Fig 5H and 5I).The reduction of CAM expression by PLC was likely responsible for the inhibition of leukocyte adhesion, that characterizes endothelial activation (Fig 5J and 5K). Similar findings were also documented in PLC-treated HUVECs (S2 Fig).

### PLC counteracts serum deprivation-induced oxidative stress

**Fig 6:**
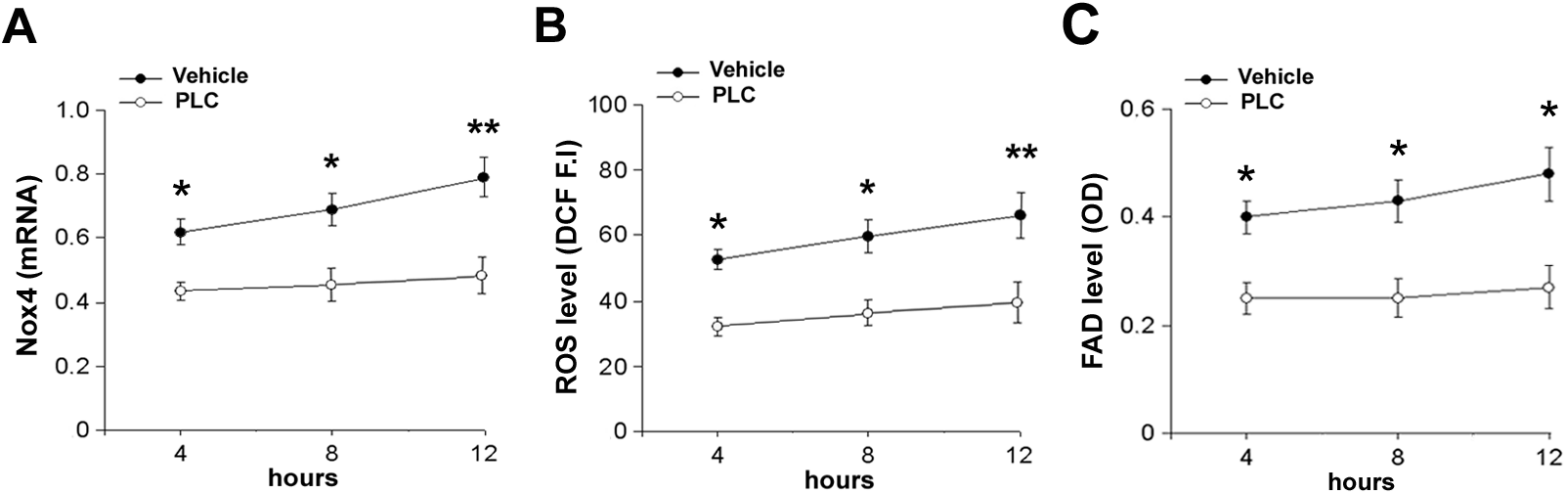
PLC reduces oxidative stress in serum-deprived HMVECs. (A) Real-time PCR for Nox4 transcripts in serum-deprived PBS-treated (vehicle) or PLC-treated HMVECs at different times. (B) ROS level detection by dichlorodihydrofluorescein fluorescence intensity (DCF F.I). (C) FAD level (β-oxidation impairment) measured as optical density (OD) assay. t-Student: * and ** indicate p< 0.05 and p< 0.01, respectively. Values are expressed as mean ± SEM of three separate experiments. Abbreviations: OD, optical density.

In HMVEC cultures, PLC treatment counteracted serum deprivation-induced oxidative stress and mitochondrial impairment, associated with endothelial dysfunction. In particular, PLC reduced Nox4 expression and ROS generation (Fig 6A and 6B). Moreover, as shown in Fig 6C, serum deprivation also determined a time-dependent increase of FAD level, indicating an impairment of β-oxidation and mitochondrial function; PLC treatment counteracted the impairment of β-oxidation. Similar findings were also documented in PLC-treated HUVEC cultures (S2 Fig).

### Serum deprivation-induced endothelial dysfunction is mediated by Nox4-dependentoxidative stress

**Fig 7:**
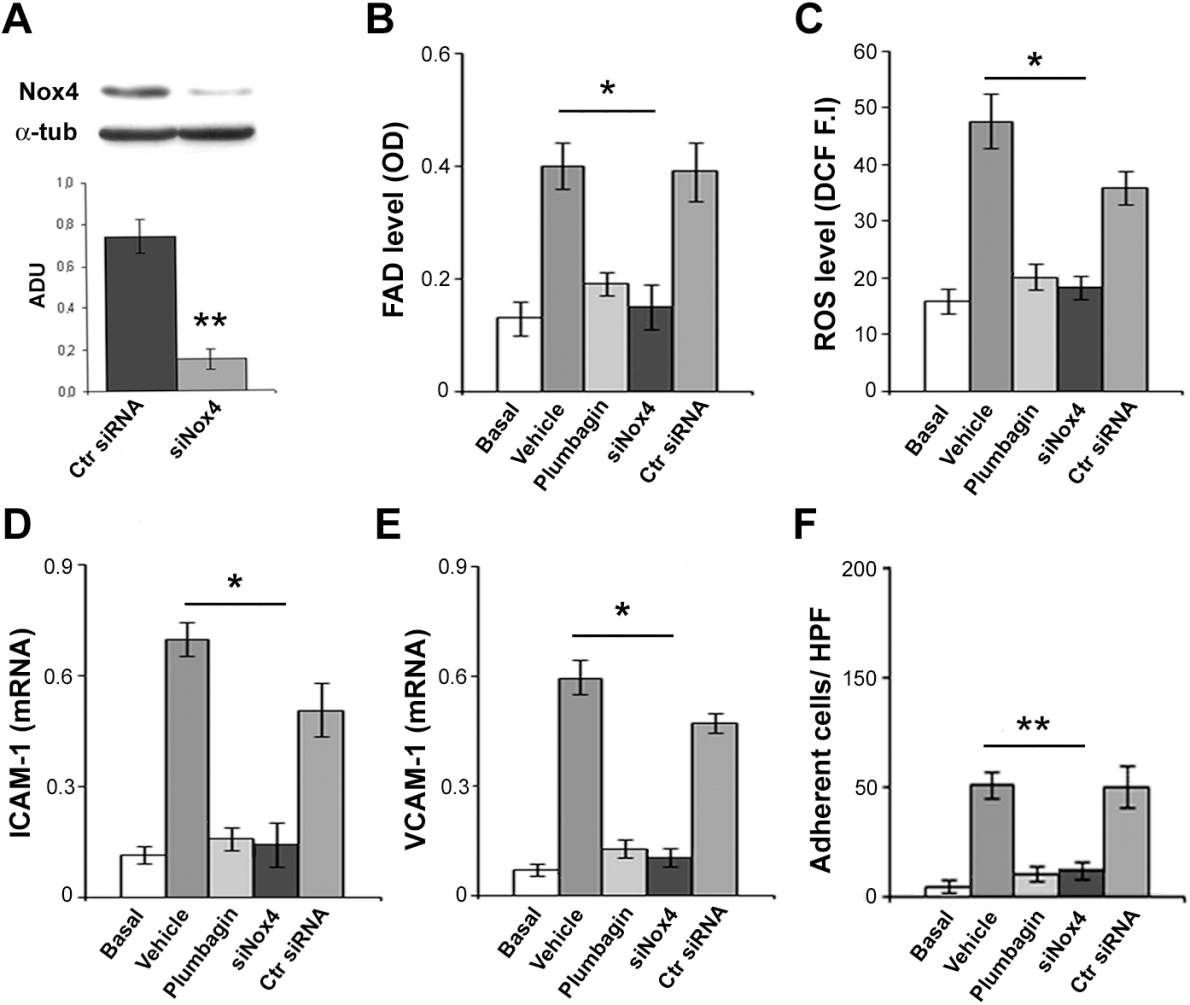
Endothelial dysfunction in serum-deprived HMVECs is mediated by NADPH oxidase 4-dependent oxidative stress. (A) Representative blot for Nox4 protein level in HMVECs transfected with non-targeting siRNA (Ctr siRNA) and siNox4. (B) FAD level (β-oxidation impairment) measured as optical density (OD) assay in basal condition (5% FBS) or serum-deprived HMVECs treated with PBS (vehicle, 12h), plumbagin (10μM in PBS, 12h), siNox4 or non-targeting siRNA (Ctr siRNA). (C) ROS level detection by dichlorodihydrofluorescein fluorescence intensity (DCF F.I). (D,E) Real-time PCR of ICAM-1 and VCAM-1 mRNA in basal, vehicle, plumbagin, siNox4 or non-targeting siRNA treated HMVECs. (F) Leukocyte adhesion assay in basal, vehicle, plumbagin, siNox4 or non-targeting siRNA treated HMVECs. t-Student: * and ** indicate p< 0.01 and p< 0.001. Values are expressed as mean ± SEM of three separate experiments. Abbreviations: OD, optical density; HPF, high power field.

To verify if Nox4 activity was responsible for oxidative stress in serum-deprived HMVECs, we used the specific Nox4 inhibitor plumbagin [38] and siRNA for Nox4 (siNox4). Specific knockdown of Nox4 was evaluated by blot analysis (Fig 7A). Plumbagin or siNox4 counteracted serum deprivation-induced oxidative stress, as FAD and ROS accumulation (Fig 7B and 7C, p< 0.0001). Plumbagin and siNox4 also prevented serum deprivation-induced increase of ICAM-1 and VCAM-1 expression (Fig 7D and 7E, p< 0.001 and p< 0.01, respectively), as well as leukocyte adhesion (Fig 7F, p< 0.001). Non-targeting siRNA (Ctr siRNA) had no effect. These findings strongly suggest that oxidative stress-induced endothelial dysfunction is mediated by Nox4 activity.

### PLC ameliorates mitochondrial β-oxidation in serum-deprived HMVEC cultures

**Fig 8:**
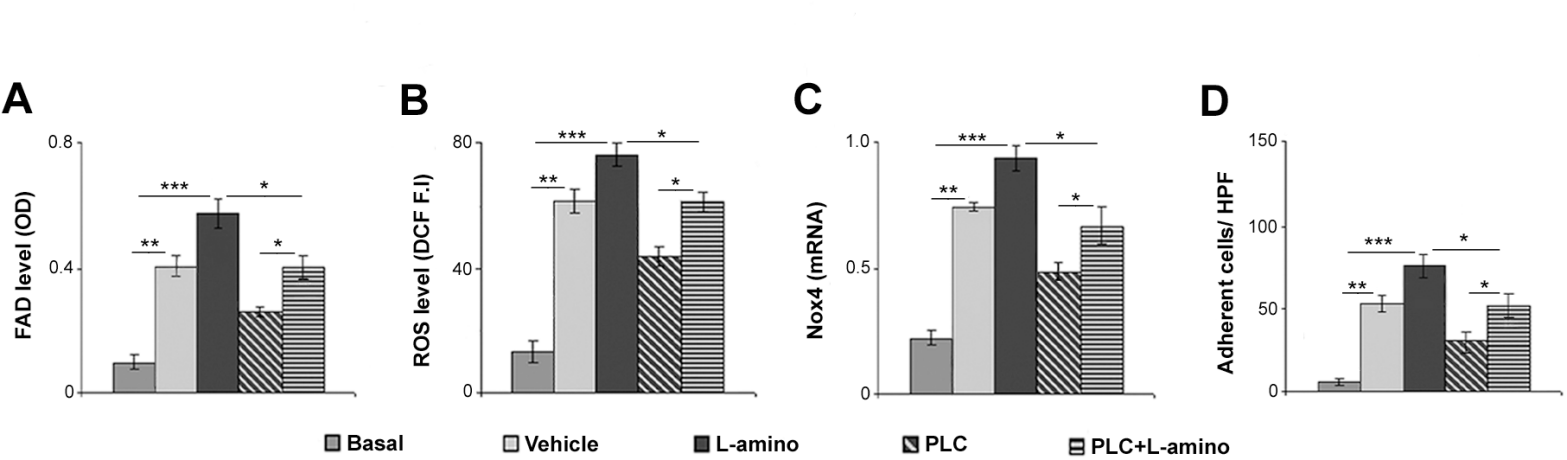
PLC ameliorates mitochondrial β-oxidation in serum-deprived HMVECs. (A) FAD level (β-oxidation impairment) measured as optical density (OD) assay in basal condition (5% FBS) and serum-deprived HMVECs treated with PBS (vehicle, 12h), L-aminocarnitine (L-amino, 1μM, 12h) and/or PLC (1mM, 12h). (B) ROS level detection by dichlorodihydrofluorescein fluorescence intensity (DCF, F.I) assay in treated cells. (C) Real-time PCR for Nox4 transcripts in treated cells. (D) Leukocyte adhesion evaluation in treated cells. Data are shown as mean ± SEM. Student’s t-test: *, ** and *** indicate p< 0.05; p< 0.01 and p< 0.001. Abbreviations: OD, optical density; HPF, high power field.

Since PLC has a specific role in the transport of fatty acids into the mitochondria [22], we investigated if β-oxidation is the pharmacological target of PLC and its regulation is involved in the endothelial oxidative stress using the specific inhibitor L-aminocarnitine [42]. As shown in Fig 8, in HMVEC cultures L-aminocarnitine induced the impairment of β-oxidation as documented by the increase of FAD level, ROS generation and Nox4 expression, as well as leukocyte adhesion (p< 0.001). Interestingly, the adding of L-aminocarnitine counteracted the antioxidant effect of PLC in serum-deprived HMVECs (p< 0.05), strongly supporting that the pharmacological target of PLC is the mitochondrial β-oxidation, that is in turn involved in Nox4-dependent oxidative stress.

## Discussion

In this study, we documented that PLC improves skin flap viability and accelerates cutaneous wound healing in vitro and in vivo. Skin flaps are frequently used in plastic and reconstructive surgery to repair large skin defects and deep wounds formed from injuries, surgery, ulcerations, or congenital defects [43], and distal skin ischemic necrosis is a well-known complication in skin flap surgery. In the skin flap, the distal segment of the flap progresses through ischemia to necrosis, so that new vessel development is required to provide blood flow [30,31]. Efficacy of PLC was confirmed on dorsal full-thickness skin wound in rats. Cutaneous wounds require, as critical component of healing, the growth of new vessels [44]. The general consensus is that unpredictable vasospasm, thrombosis, and insufficient vascular supply mediates skin flap ischemic necrosis [45]. Pharmacological treatment to enhance skin flap viability by promoting local angiogenesis and, in turn, augmenting skin flap blood flow and viability has been the focus of much research in the past two decades. The PLC dosage was chosen in the range to be effective in protecting endothelial cells and in increasing rat plasma NO level [46,47]. In the model of skin flap, new developing vessels are described to provide blood flow to the ischemic skin flap mimicking the clinical process of angiogenesis in reconstructive surgery [28,31]. The extent of skin flap survival is directly correlated to the development of new vessels and considered its direct measurement [28]. PLC has been recently introduced among emerging non-interventional medical regiments that aim to counteract peripheral artery disease-related adverse effects [48]. PLC accelerates blood-flow recovery and the restoration of vascular function in a rabbit model of hind limb ischemia by sustaining vasodilatative and arteriogenic remodeling [24]. The faster recovery of blood perfusion and the increased flap viability observed in PLC-treated rats suggest that PLC supports vasodilatative effects and a more rapid formation of new small vessels. Although not fully representative of chronic ulcers observed in human subjects, full-thickness skin wound or skin flap necrosis share with ischemic ulcers some features and neovascularization as critical factor in the healing process [28,49]. Immunohistochemistry demonstrated in PLC-treated rat skin granulation tissue microvessels the increase of CD31^+^ capillaries and iNOS, VEGF and PlGF expression, in line with a neoangiogenic and vasodilatative effect of PLC. In fact, PLC treatment is known to induce also vasodilatation in isolated arterioles and in arteries after experimental ligation [24,50], although vasodilatation was not associated with modification of tissue capillary density of ischemic tissues [24] as we documented here in the dermal granulation tissue. These results are in agreement with previous data showing that increased iNOS activity promotes ischemic skin flap survival by enhancing development of new vessels [28]. NO is a potent vasodilator released by endothelial cells, and dilatation of preexisting vessels plays a major role in early adaptation favoring flow recovery [51]. iNOS activity has been documented to favor skin wound repair and significant increase of iNOS activity occurs in wound process, associated with an up-regulated production of VEGF [52]. The iNOS level has also been positively correlated to collagen deposition in skin wounds [53]. The maintenance of vascular function is essential to support circulation integrity, and therefore, homeostasis of tissue environments under physiological conditions [54]. Previous reports described a positive effect of PLC vascular remodeling after mechanical and atherogenic injury [55], in part reducing the recruitment and number of plaque cells by apoptosis [55,56]. Vasodilatation is an early and NO-mediated process, that precedes angiogenesis [57]. In fact, we reported a PLC-induced increase of PlGF, iNOS expression and NO level in HMVEC cultures. NO production by iNOS induction modulates the release of growth factors, including VEGF and PlGF, that play a critical role in new vessel formation [58–60]. PlGF is a particularly interesting member of the VEGF family because of its predominantly arteriogenic effects and its ability to release endothelial progenitor cells from the bone marrow [61]. In addition, the increase of PlGF represents a well-known mechanism that stimulates angiogenesis for its preferential interaction with Flt-1 [62]. Flt-1 and KDR expression were similarly up-regulated by PLC in HMVEC cultures.

Experimental data suggest that wound healing associates with endothelial aberrations suggestive of localized dysfunction [3]. Several noxious stimuli, including diabetes, dyslipidemia, and oxidative stress, induce endothelial dysfunction [63]. ROS family includes molecular oxygen and its derivatives produced in all aerobic cells. Excessive vascular production of ROS, outstripping endogenous antioxidant defence mechanisms, has been implicated in oxidation of biological macromolecules, such as DNA, protein, carbohydrates, and lipids [64]. This condition has commonly been referred as oxidative stress [64]. Inflammatory stimuli increase cellular oxidative stress driven by mitochondrial and Nox-dependent ROS generation [13,14]; in endothelial cells, oxidative stress causes mitochondrial dysfunction and impairment of β-oxidation [15,16]. The evidence of an interplay between mitochondrial and NADPH oxidase-derived ROS constitutes a feed-forward cycle in which mitochondrial ROS increase NADPH oxidase-dependent ROS production, that in turn increases mitochondrial ROS generation, in a sort of vicious cycle [14]. Oxidative stress driven by mitochondrial and/or Nox-mediated ROS generation activates the downstream-regulated inflammatory response of endothelial cells [16,59,65], inducing de novo CAM expression and secretion of inflammatory cytokines responsible for leukocyte recruitment and adhesion [9]. In PLC-treated skin flaps, ROS production was reduced and iNOS and NO level increased. Immunohistochemistry performed on rat skin granulation tissue documented reduced Nox4^+^ microvessels with PLC treatment; in addition, PLC in HMVEC cultures reduced serum deprivation-induced Nox4 expression and ROS generation, mitochondrial β-oxidation impairment, CAM expression and leukocyte adhesion; the latter normally characterize endothelial activation [18,19]. Moreover, the specific inhibition of Nox4 by plumbagin or siRNA counteracted oxidative stress and leukocyte adhesion, supporting that serum deprivation-induced endothelial activation is Nox4-dependent. Our in vitro studies in HMVEC cultures demonstrated that beneficial effects of PLC depends on its activity on mitochondrial β-oxidation. In fact, the inhibition of β-oxidation counteracted beneficial effects of PLC on serum deprivation-induced oxidative stress and endothelial activation, confirming that the pharmacological target of PLC is the mitochondrial β-oxidation, that is in turn involved in Nox4-dependent oxidative stress. These findings support the hypothesis that the antioxidant properties of PLC play a role in the amelioration of vascular function [8,24]. Additional studies may be extended to vascular smooth muscle and/or progenitor cells to assess the effects of antioxidant treatment in the progression of atherosclerosis and in the arterial response to damage, including hypoxia and aging-induced remodeling [66–68]. A the present, given the beneficial effects of PLC on angiogenesis, potential limitations for its clinical employment must be considered, in particular in cancer patients, where increased tumor angiogenesis is considered a unfavorable factor [69]. Further studies are needed to investigate the effects of PLC on tumor angiogenesis in vivo and in vitro, although same report documented a beneficial effect of carnitine derivatives in preclinical models of cancer progression [70].

In conclusion, PLC treatment enhanced vasodilatation and angiogenesis in skin flap and accelerates cutaneous wound healing. Molecular data from the in vitro studies suggest that the beneficial effects of PLC are due to its antioxidant and anti-inflammatory activity on endothelial cells. These findings support the therapeutic use in healthy patients of PLC and, more in general, of antioxidant agents and the targeting of endothelial dysfunction in the management of acute and chronic wounds in clinical settings.

## Supporting Information

S1 Fig PLC reduces NADPH oxidase 4-dependent oxidative stress in serum-deprived HMVECs and ameliorates mitochondrial β-oxidation. (A) ROS level detection by dichlorodihydrofluorescein fluorescence intensity (DCF F.I) on adherent cells in the 96-well plate in serum-deprived PBS-treated (vehicle) or PLC-treated HMVECs at different times. (B) ROS level in basal condition (5% FBS) or serum-deprived HMVECs treated with PBS (vehicle, 12h), plumbagin (10μM in PBS, 12h), siNox4 or non-targeting siRNA (Ctr siRNA). (C) ROS level in basal condition (5% FBS) and serum-deprived HMVECs treated with PBS (vehicle, 12h), L-aminocarnitine (L-amino, 1μM, 12h) and/or PLC (1mM, 12h). t-Student: *, ** and *** indicate p< 0.05; p< 0.01 and p< 0.001, respectively. Values are expressed as mean ± SEM of three separate experiments.
(TIF)

S2 Fig Effects of PLC stimulation on human umbilical vein endothelial cells (HUVECs). (A-C) Real-time PCR for eNOS, iNOS and PlGF mRNA in serum-deprived PBS-treated (vehicle) or PLC-treated HUVECs at different times. (D) PlGF protein concentration assessed by means of ELISA. (E-F) Real-time PCR for VCAM-1 and ICAM-1 mRNA in treated cells. (G) FAD level (β-oxidation impairment) measured as optical density (OD) assay (4h-treatment). (H) Real-time PCR for Nox4 transcripts in treated cells (4h-treatment). (I) ROS level detection by dichlorodihydrofluorescein fluorescence intensity (DCF F.I.) in vehicle or PLC-treated cells after (4h-treatment). (J) Leukocyte adhesion assay on vehicle and 4h PLC-treated HUVECs. t-Student: * and ** indicate p< 0.05 and p< 0.01, respectively. Values are expressed as mean ± SEM of three separate experiments. Abbreviations: OD, optical density; HPF, high power field.
(TIF)
