# Supplementary figures and images for: Propionyl-L-Carnitine Enhances Wound Healing and Counteracts Microvascular Endothelial Cell Dysfunction
Source: PLoS One. 2015 Oct 16;10(10):e0140697. doi: 10.1371/journal.pone.0140697 (PMC4608702; doi:10.1371/journal.pone.0140697)

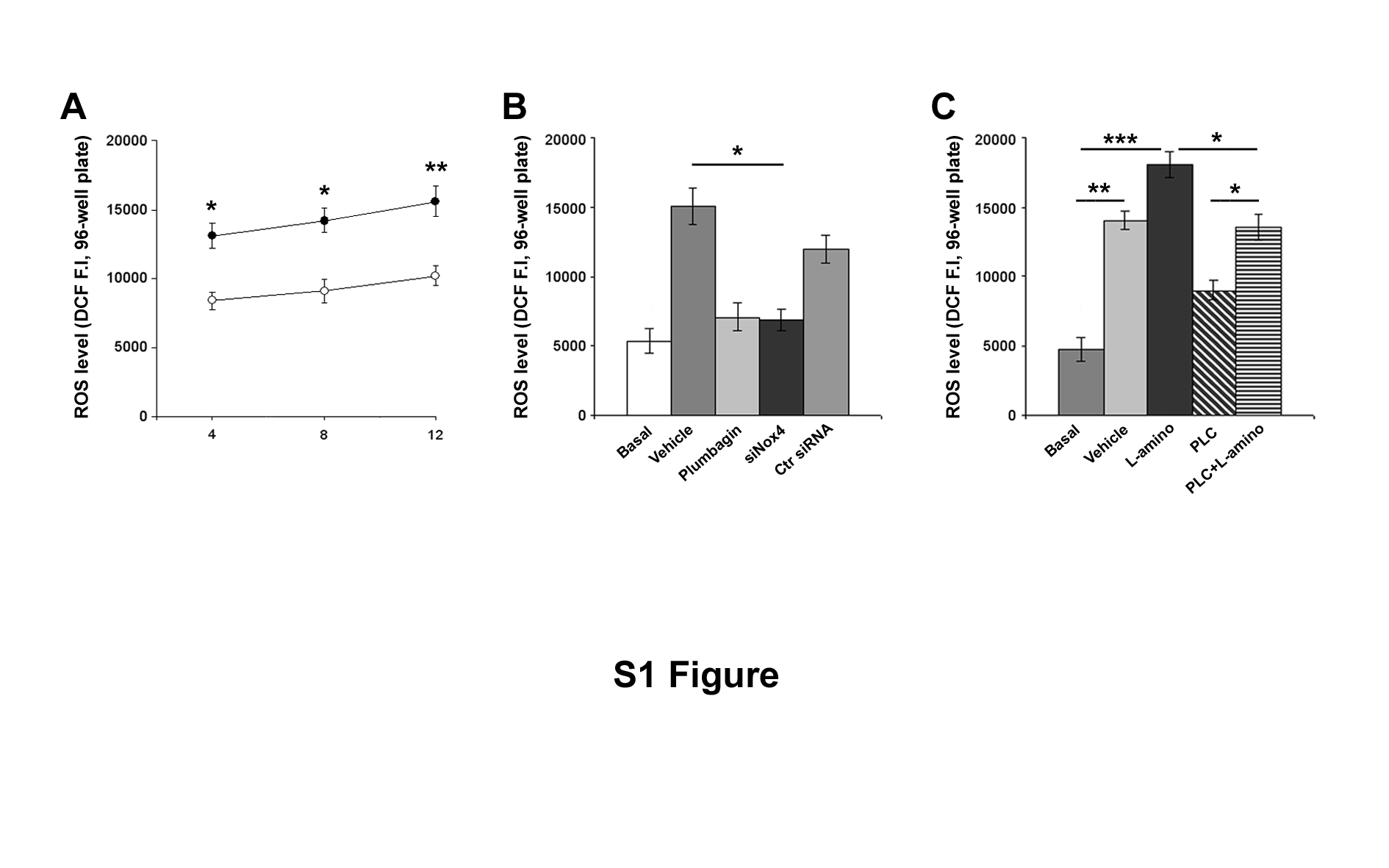

Supplement: S1 Fig — (A) ROS level detection by dichlorodihydrofluorescein fluorescence intensity (DCF F.I) on adherent cells in the 96-well plate in serum-deprived PBS-treated (vehicle) or PLC-treated HMVECs at different times. (B) ROS level in basal condition (5% FBS) or serum-deprived HMVECs treated with PBS (vehicle, 12h), plumbagin (10μM in PBS, 12h), siNox4 or non-targeting siRNA (Ctr siRNA). (C) ROS level in basal condition (5% FBS) and serum-deprived HMVECs treated with PBS (vehicle, 12h), L-aminocarnitine (L-amino, 1μM, 12h) and/or PLC (1mM, 12h). t-Student: *, ** and *** indicate p< 0.05; p< 0.01 and p< 0.001, respectively. Values are expressed as mean ± SEM of three separate experiments. (TIF) [file pone.0140697.s001.tif]

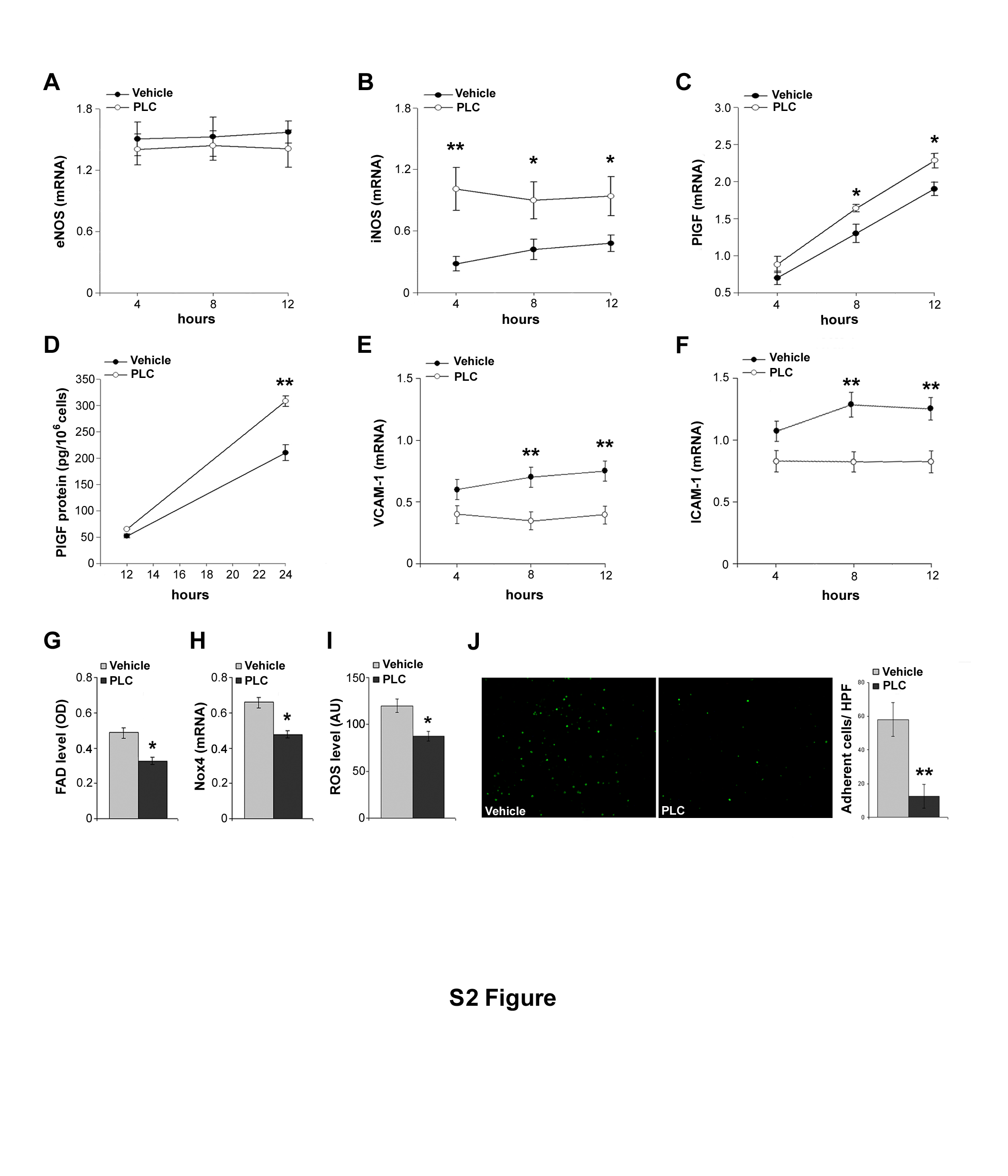

Supplement: S2 Fig — (A-C) Real-time PCR for eNOS, iNOS and PlGF mRNA in serum-deprived PBS-treated (vehicle) or PLC-treated HUVECs at different times. (D) PlGF protein concentration assessed by means of ELISA. (E-F) Real-time PCR for VCAM-1 and ICAM-1 mRNA in treated cells. (G) FAD level (β-oxidation impairment) measured as optical density (OD) assay (4h-treatment). (H) Real-time PCR for Nox4 transcripts in treated cells (4h-treatment). (I) ROS level detection by dichlorodihydrofluorescein fluorescence intensity (DCF F.I.) in vehicle or PLC-treated cells after (4h-treatment). (J) Leukocyte adhesion assay on vehicle and 4h PLC-treated HUVECs. t-Student: * and ** indicate p< 0.05 and p< 0.01, respectively. Values are expressed as mean ± SEM of three separate experiments. Abbreviations: OD, optical density; HPF, high power field. (TIF) [file pone.0140697.s002.tif]
